# Supplementary material for: A Machine-Learning Method to Assess Growth Patterns in Plants of the Family Lemnaceae
Source: Plants (Basel). 2022 Jul 23;11(15):1910. doi: 10.3390/plants11151910 (PMC9332063; doi:10.3390/plants11151910)
Supplement: Supplementary file 1 [file plants-11-01910-s001.zip › Frond area evaluation in Fiji.pdf]

Frond area evaluation in Fiji

To evaluate the frond area in Fiji, we have adopted the following protocol:

1. Open the picture in Fiji
2. Prepare the image so that nothing interferes with the measuring, e.g. reflections or other artefacts
3. Image > Adjust > Color threshold
4. In the color threshold window, move the indicator bar of the Hue channel to select the green color
5. In the same window, Adjust saturation and Brightness so that fronds become completely red
6. Now press "select" to select the highlighted red area
7. Analyze > Measure
8. The software will output data expressed in pixel
9. Transfer data into Excell
