# Supplementary material for: A Machine-Learning Method to Assess Growth Patterns in Plants of the Family Lemnaceae
Source: Plants (Basel). 2022 Jul 23;11(15):1910. doi: 10.3390/plants11151910 (PMC9332063; doi:10.3390/plants11151910)

Protocol to analyze Lemanacae area with ilastik software. Futherinformation can be foundf on the official page of the ilastik software: https://www.ilastik.org/documentation/objects/objects

1) Transform images from Jpeg or other formats to TIFF
2) Open ilastik
3) Select : pixel classification + object classification
4) Input data (select the picture with the highest number of fronds)

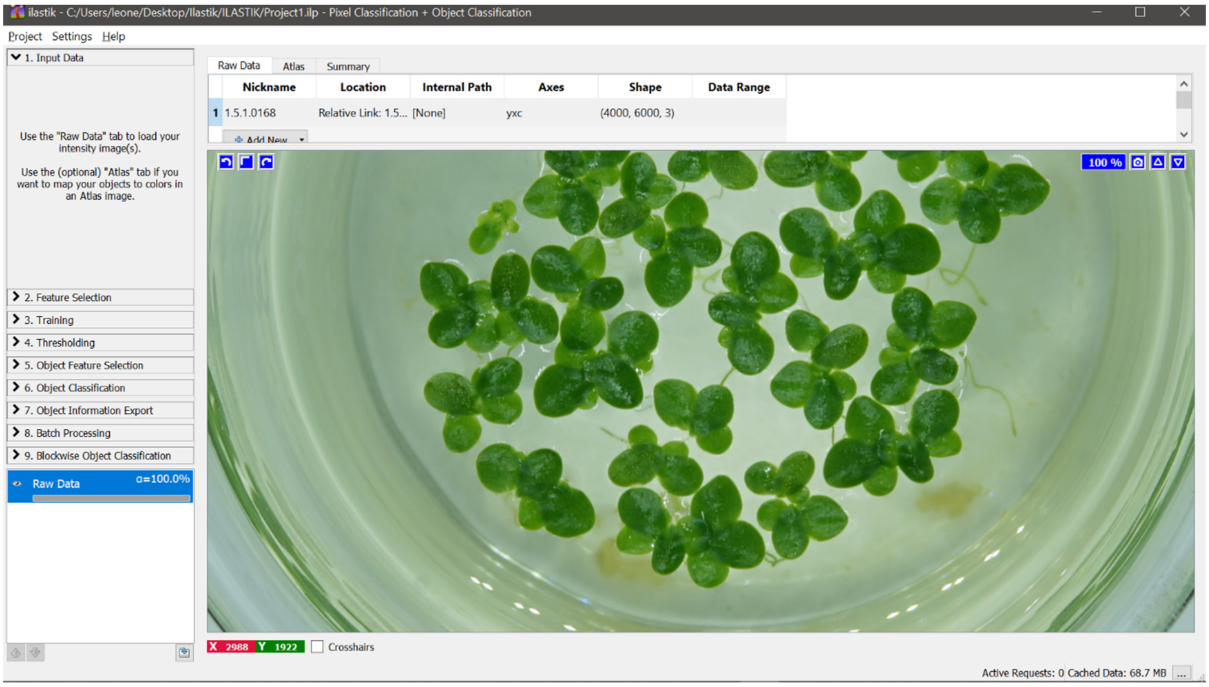

5) Feature selection: select αx to αy (note that the higher the number of selected features, the heavier the computational calculation will be in the following steps)

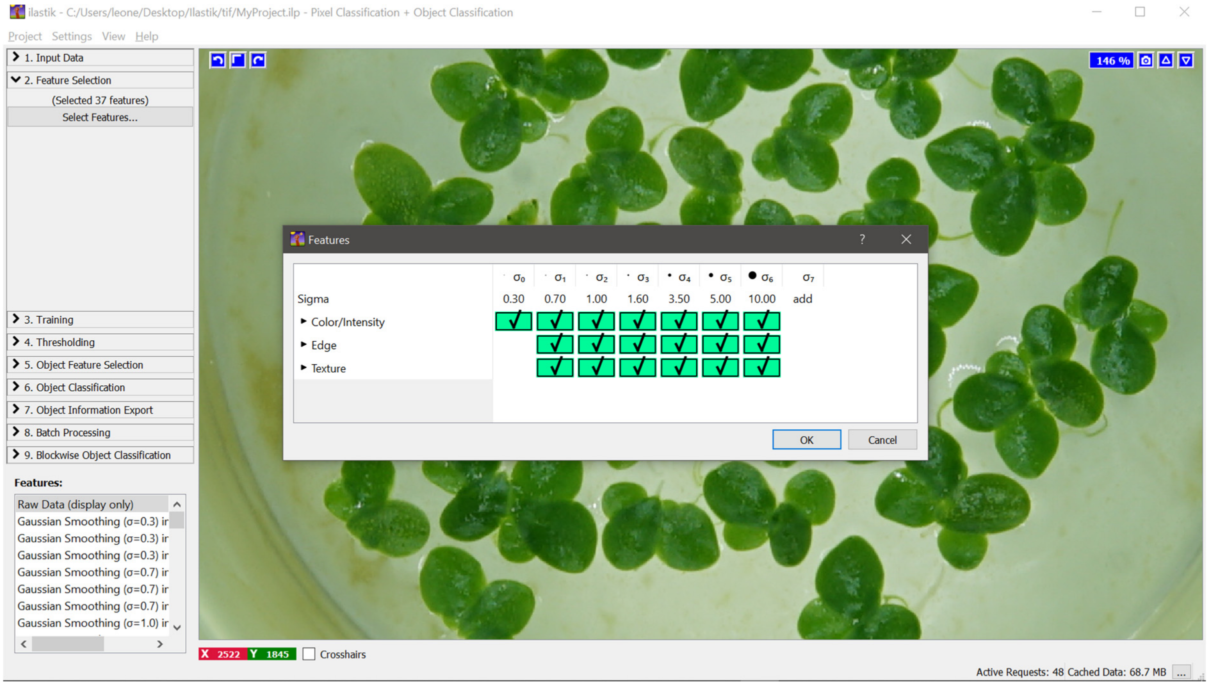

6) Training, rename labels by double clicking.

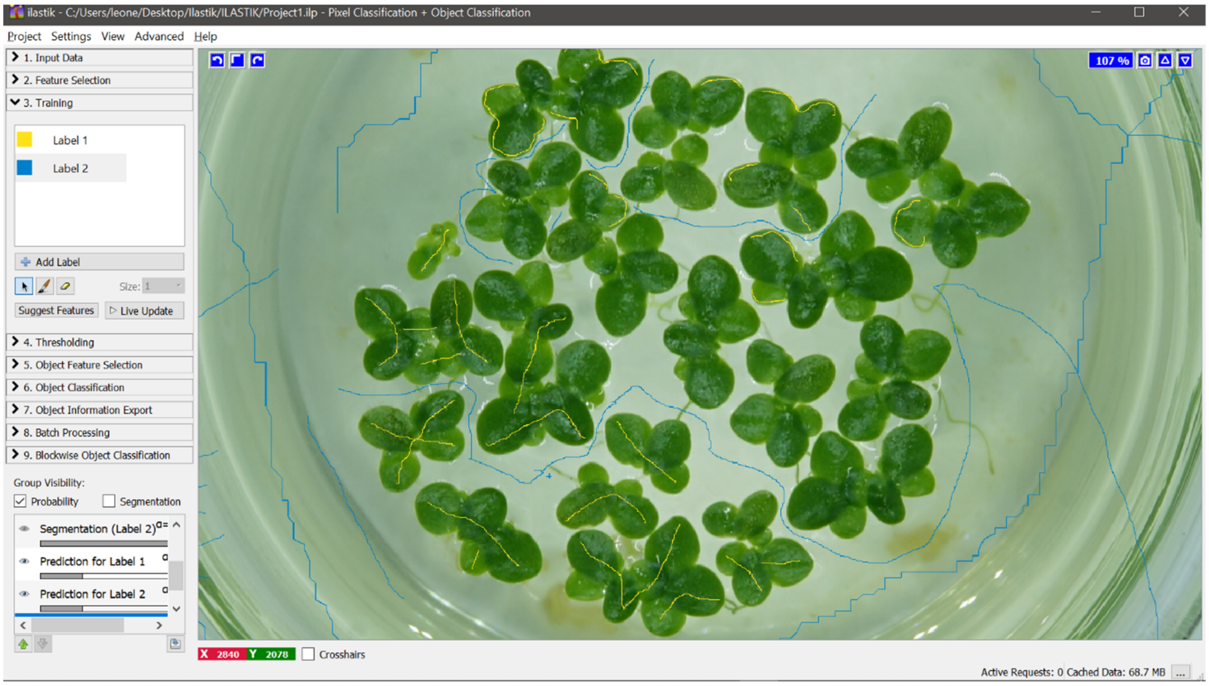

7) Suggest features
8) Feature selection (choose the most appropriate to the selected once)
9) Run feature selection
10) Select feature set
11) Apply Thresholding (standard)

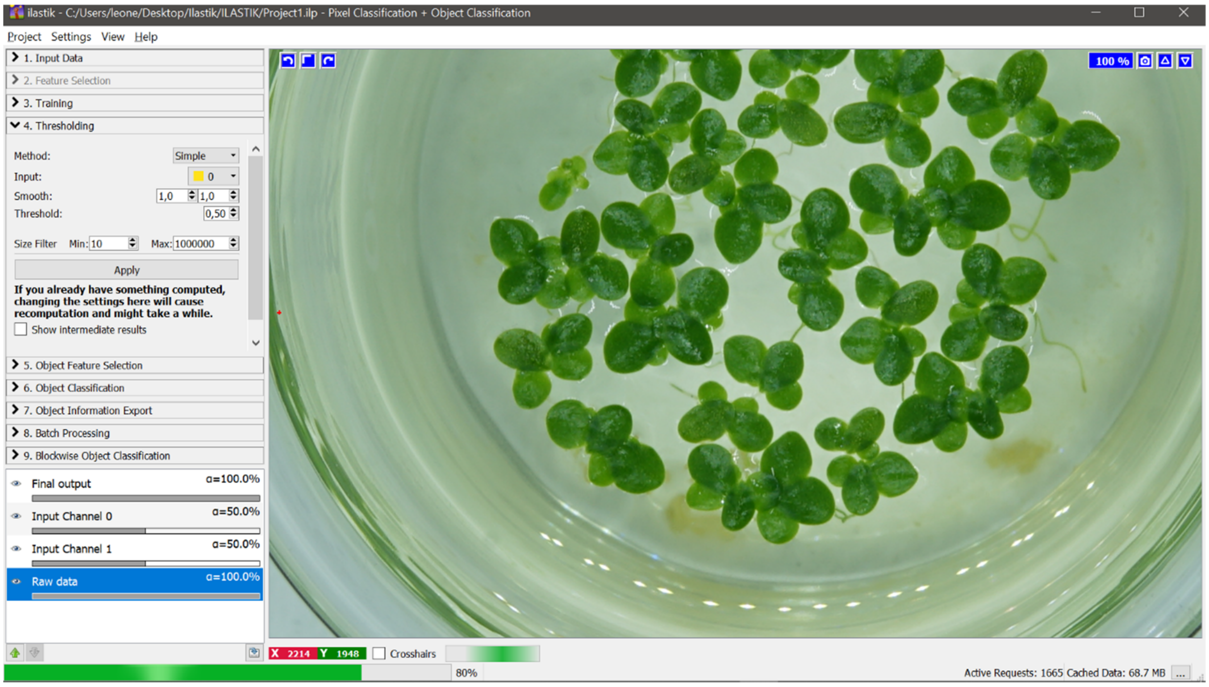

12) Object feature selection:

- Select feature: Size in pixle

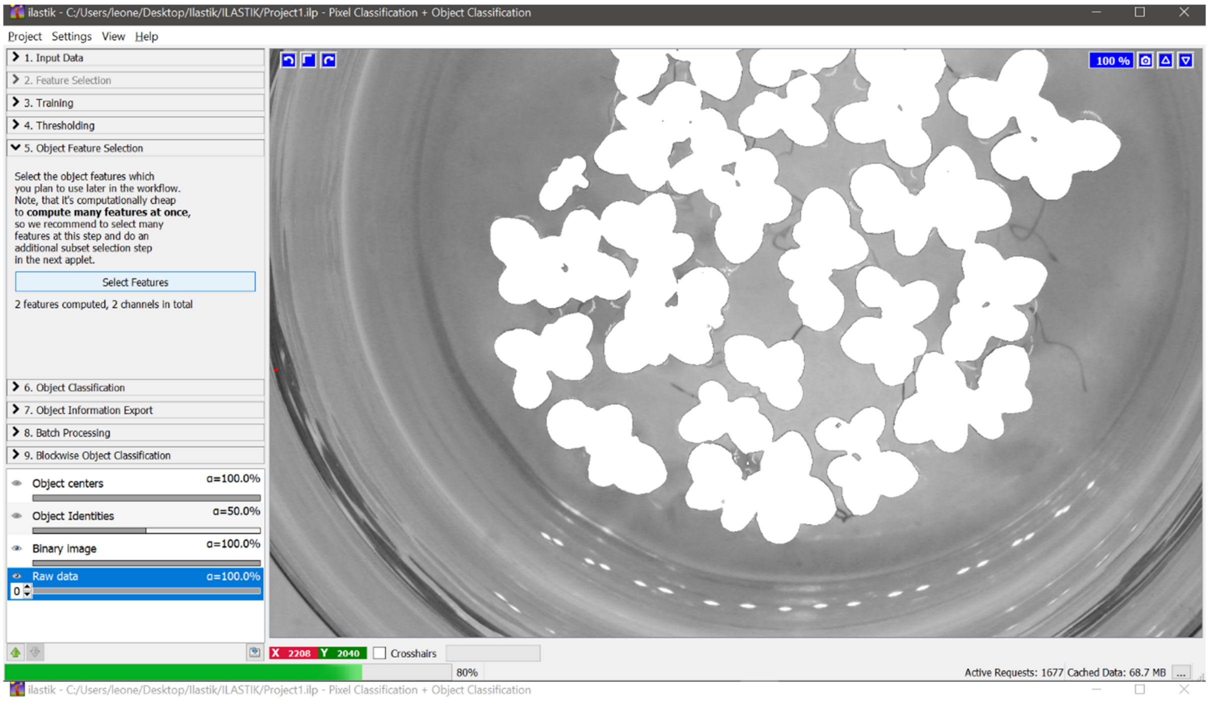

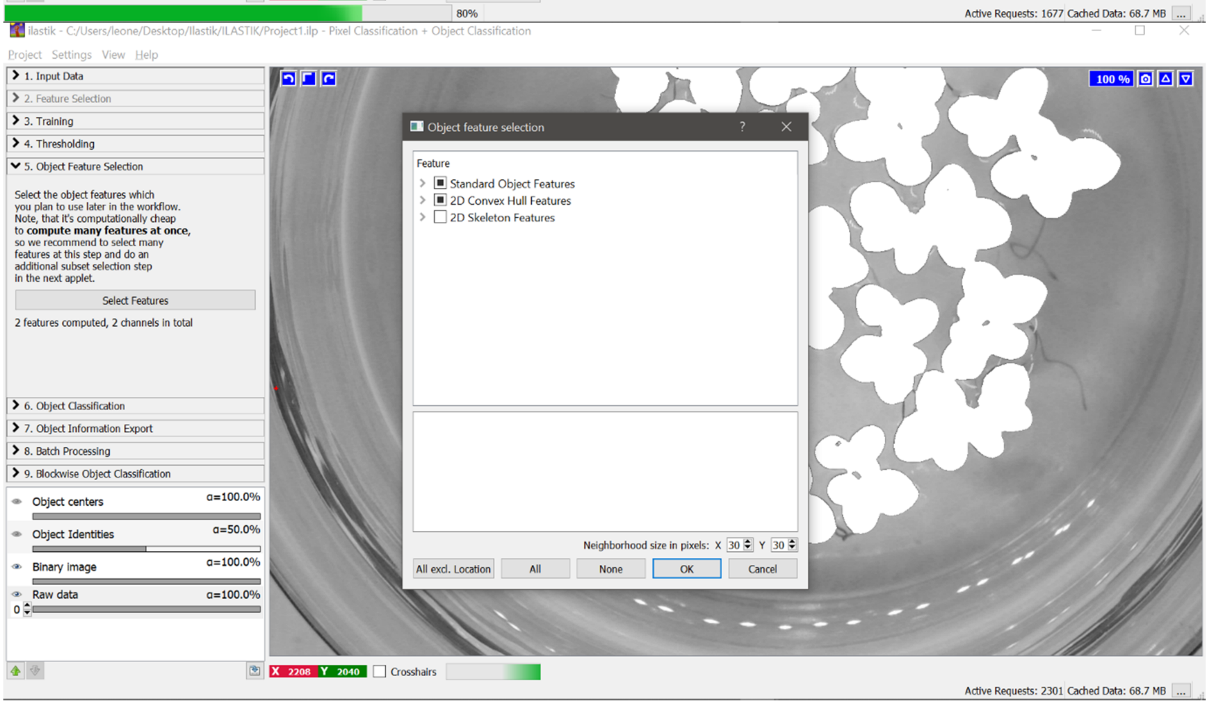

13) Object classification: add lable to the white selection

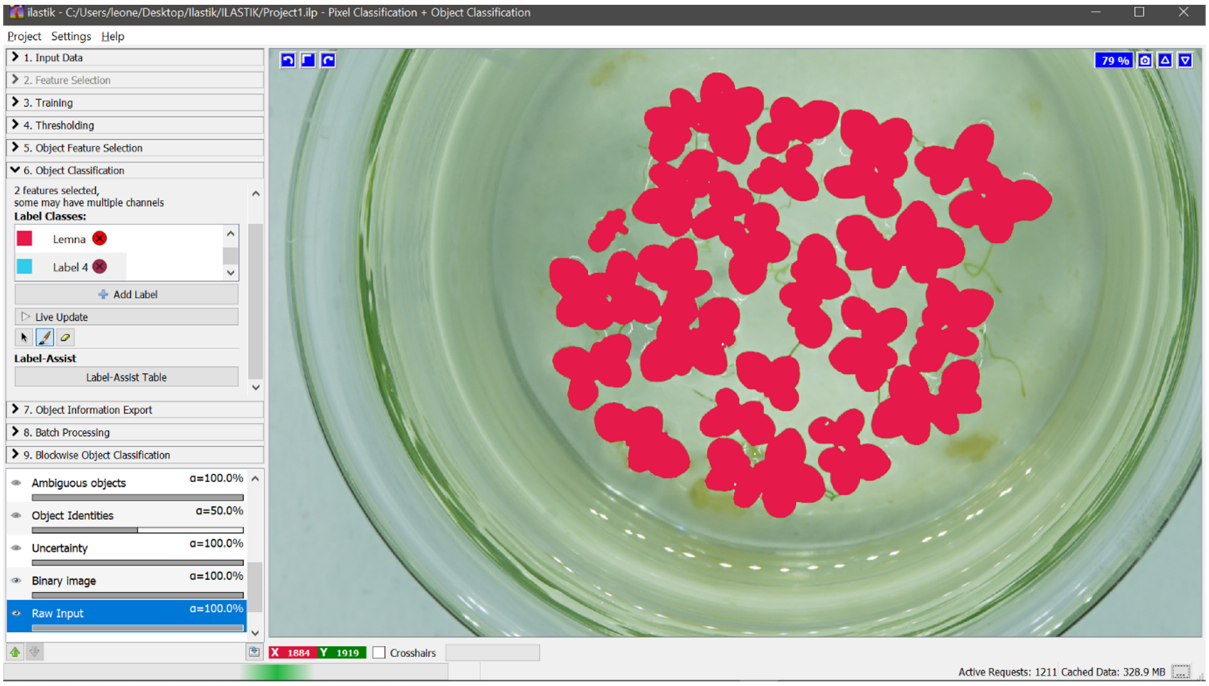

14) Object information export

- Source: object probabilities
- Choose export image setting: format .tif
- Configure table export: Format .csv

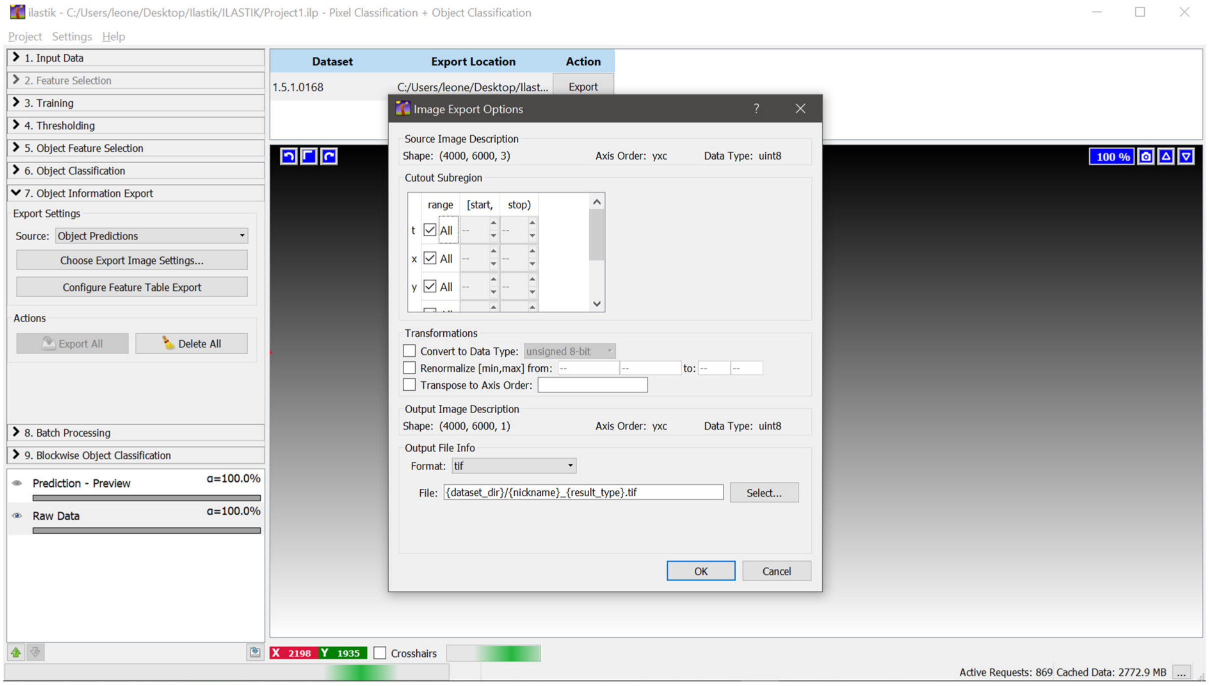

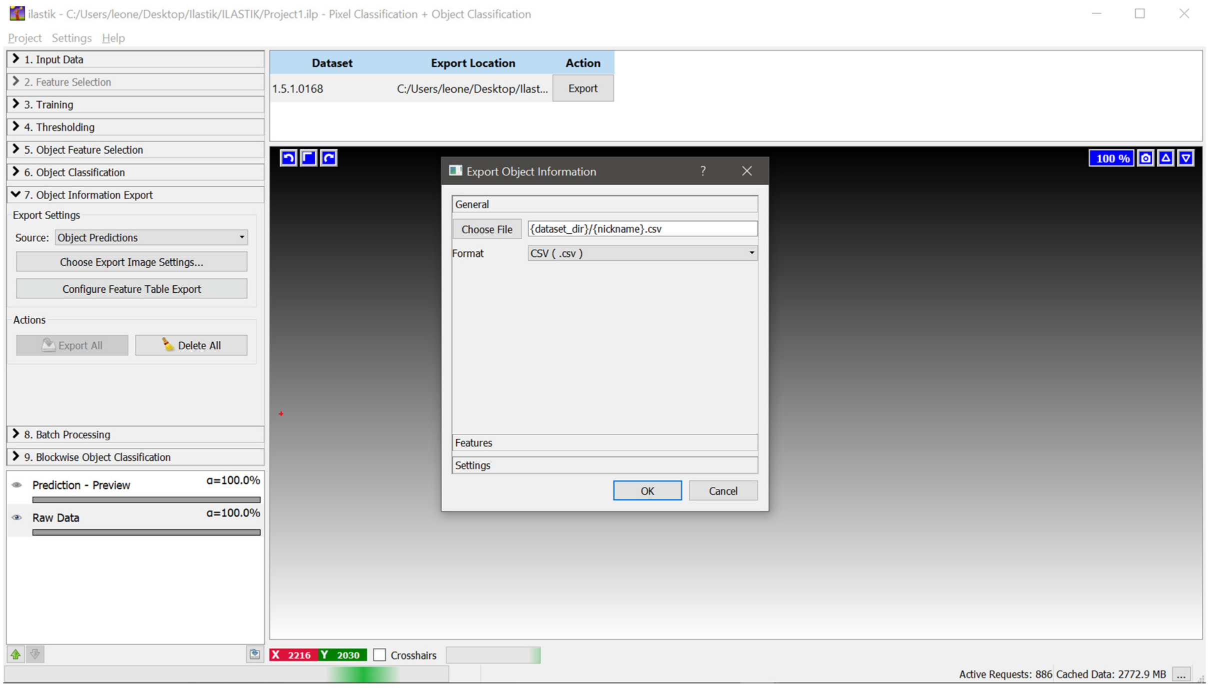

15) Batch processing
16) Process all files
17) Output

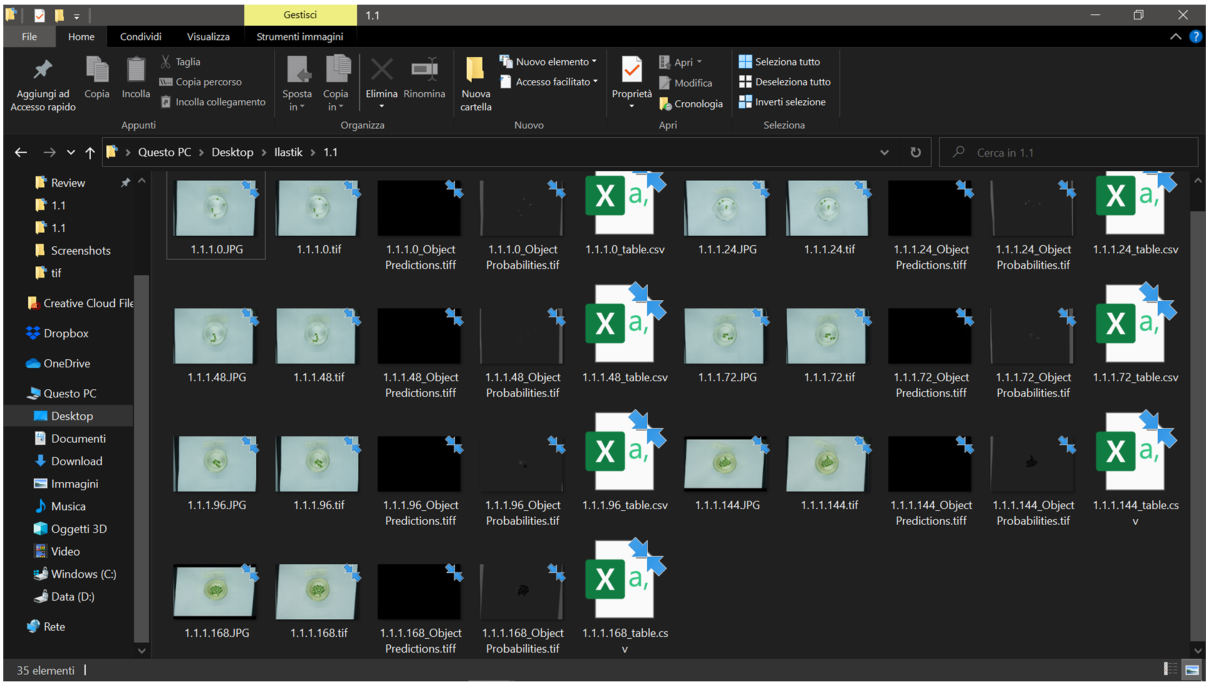

Supplement: Supplementary file 1 [file plants-11-01910-s001.zip › ilastik_protocol.pdf]
